# PV1, a novel *Plasmodium falciparum* merozoite dense granule protein, interacts with exported protein in infected erythrocytes

**DOI:** 10.1038/s41598-018-22026-0

**Published:** 2018-02-27

**Authors:** Masayuki Morita, Hikaru Nagaoka, Edward H. Ntege, Bernard N. Kanoi, Daisuke Ito, Takahiro Nakata, Ji-Won Lee, Kazuaki Tokunaga, Tadahiro Iimura, Motomi Torii, Takafumi Tsuboi, Eizo Takashima

**Affiliations:** 1 Division of Malaria Research, Proteo-Science Center, Ehime University, Matsuyama, Ehime Japan; 2 Division of Bio-Imaging, Proteo-Science Center, Ehime University, Shitsukawa, Toon, Ehime Japan; 3 Nikon Instech CO., LTD., Shinagawa, Tokyo Japan; 4 Division of Analytical Bio-Medicine, Advanced Research Support Center, Ehime University, Shitsukawa, Toon, Ehime Japan; 5 Division of Molecular Parasitology, Proteo-Science Center, Ehime University, Shitsukawa, Toon, Ehime Japan; 6 Present Address: Division of Medical Zoology, Department of Microbiology and Immunology, Faculty of Medicine, Tottori University, Yonago, Tottori, 683-8503 Japan

## Abstract

Upon invasion, *Plasmodium falciparum* exports hundreds of proteins across its surrounding parasitophorous vacuole membrane (PVM) to remodel the infected erythrocyte. Although this phenomenon is crucial for the parasite growth and virulence, elucidation of precise steps in the export pathway is still required. A translocon protein complex, PTEX, is the only known pathway that mediates passage of exported proteins across the PVM. *P*. *falciparum* Parasitophorous Vacuolar protein 1 (PfPV1), a previously reported parasitophorous vacuole (PV) protein, is considered essential for parasite growth. In this study, we characterized PfPV1 as a novel merozoite dense granule protein. Structured illumination microscopy (SIM) analyses demonstrated that PfPV1 partially co-localized with EXP2, suggesting the protein could be a PTEX accessory molecule. Furthermore, PfPV1 and exported protein PTP5 co-immunoprecipitated with anti-PfPV1 antibody. Surface plasmon resonance (SPR) confirmed the proteins’ direct interaction. Additionally, we identified a PfPV1 High-affinity Region (PHR) at the C-terminal side of PTP5 where PfPV1 dominantly bound. SIM analysis demonstrated an export arrest of PTP5ΔPHR, a PTP5 mutant lacking PHR, suggesting PHR is essential for PTP5 export to the infected erythrocyte cytosol. The overall results suggest that PfPV1, a novel dense granule protein, plays an important role in protein export at PV.

## Introduction

Malaria, caused by protozoan parasite *Plasmodium falciparum*, is a leading cause of morbidity and mortality in endemic regions^1^. Upon invasion, the parasite exports hundreds of proteins through its surrounding parasitophorous vacuole membrane (PVM) into the cytosol of the host erythrocyte^2^. Through this process, the infected erythrocyte is modified with changes that are crucial to the parasite’s survival and virulence^3^. *P*. *falciparum* infected erythrocytes have increased rigidity and adhere to endothelial cells (cytoadherence) in the microcirculation of various organs. This phenomenon is believed to aid the parasite evade the host immune system, and contributes to malaria pathogenesis^3^.

In order to traverse the PVM, exported proteins must be initially unfolded^4^. A translocon protein complex, *Plasmodium* translocon of exported proteins (PTEX), mediates the unfolding and passage of the parasite proteins^5–7^. To date, five PTEX constituent proteins have been characterized including; EXP2, HSP101, PTEX150, PTEX88 and TRX2^5^. EXP2, HSP101 and PTEX150 constitute the core PTEX, whereas PTEX88 and TRX2 are thought to play accessory roles^8–10^. *P*. *falciparum* Parasitophorous Vacuolar protein 1 (PfPV1; PF3D7_1129100), a previously reported *bona fide* PV protein is essential for the parasite growth^11^. A recent report indicates that PfPV1 co-precipitates with PTEX complex^12,13^, suggesting that PfPV1 constitutes PTEX accessory molecules^13^.

Cytoadherence of infected erythrocytes to microvasculature is mediated by members of the *P*. *falciparum* Erythrocyte Membrane Protein-1 (PfEMP1) family^14^. The PfEMP1 proteins are trafficked through cytosol to the surface of host erythrocytes^15^. A large-scale gene knockout study identified several exported proteins that play important roles in PfEMP1 trafficking^16^. PfEMP1-trafficking protein (PTP5) also known as PF70 (PlasmoDB PF3D7_1002100) is among the exported proteins essential for PfEMP1 trafficking^16,17^.

Most exported proteins, including PTP5, possess a *Plasmodium* export element (PEXEL) motif (RxLx/E/D/Q) at the N-terminus^18,19^. The exported proteins are proteolytically cleaved by Plasmepsin V (PMV) at the C-terminal side of the conserved leucine in the PEXEL motif followed by new N-terminal acetylation (Ac-xE/Q/D)^20^. There are approximately 500 proteins with PEXEL motif, representing ~10% of parasite genome, and are predicted to be exported proteins^4,18,19,21^. However, several exported proteins, named PEXEL-negative exported proteins (PNEPs), contain neither a PEXEL motif nor other conserved export sequences. PNEPs are a rather small group in *P*. *falciparum* and include proteins such as ring-exported proteins 1 and 2 (REX1, REX2)^22,23^, skeleton binding protein 1 (SBP1)^24^, and PfEMP1^21^. Reported knockdown studies of PTEX150 and HSP101 suggest that PTEX plays a role in the export of both PEXEL and PNEPs proteins^6,7^.

PTEX core proteins, EXP2 and PTEX150, are stored in merozoite dense granules, with subsequent translocation to the PVM upon invasion^8^. Moreover, another protein, Ring-infected Erythrocyte Surface Antigen, (RESA), is stored in the dense granules but translocated to the sub-plasma membrane region of infected erythrocyte^25^. Therefore, it could be considered that proteins localized to the dense granules might be involved in protein export in infected erythrocytes.

In this study, we have characterized PfPV1 as a novel merozoite dense granule protein. We observed that PfPV1 partially co-localizes with EXP2, suggesting that the protein could be a PTEX accessory molecule. PfPV1 and the exported protein PTP5 co-immunoprecipitated with anti-PfPV1 antibody. Surface plasmon resonance (SPR) confirmed the proteins’ direct interaction. Moreover, we identified a PfPV1 High-affinity Region (PHR) at the C-terminal side of PTP5 where PfPV1 dominantly bound. We demonstrated the arrest of PTP5ΔPHR export in PV, suggesting that PHR is essential for PTP5 export. The overall results suggest that PfPV1 a novel dense granule protein that plays an important role in protein export at PV.

## Results

### Production of recombinant PfPV1 protein

**Figure 1:**
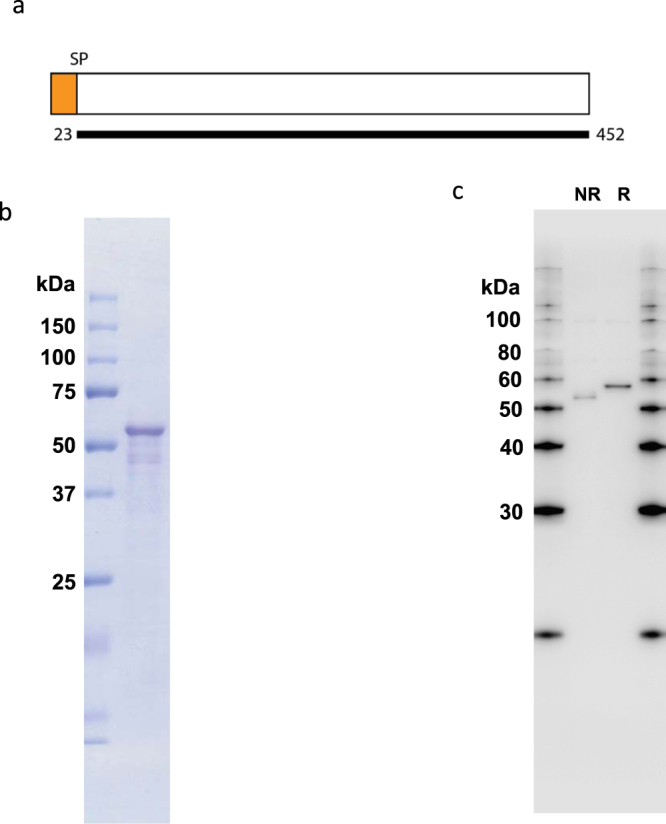
Assessment of expressed recombinant PfPV1, and rabbit anti-PfPV1 antibody. (**a**) Schematic presentation of PfPV1. The thick black line indicates region of protein expressed using WGCFS; SP is the predicted signal peptide. (**b**) Purified recombinant PfPV1 resolved in SDS-PAGE under reduced conditions and stained with Coomassie brilliant blue (CBB), (**c**) Western blot analysis of PfPV1 in trophozoite and schizont-rich parasite lysate using rabbit anti-PfPV1 antibody. NR and R indicate results of non-reduced and reduced conditions respectively.

PfPV1 (PF3D7_1129100) is predicted as a 51.9 kDa protein (Fig. 1a). Signal peptide was predicted at the 1–22 amino acid residues (aa). The protein has neither PEXEL motif nor predicted functional domain. PfPV1 orthologs were identified in various plasmodium species including, *P*. *vivax*, *P*. *knowlesi*, *P*. *chaubaudi* and *P*. *berghei*^11^. Previous studies suggested that PfPV1 is essential for blood-stage parasite growth and localizes to PV^11,26^. In order to characterize the protein further, we utilized wheat germ cell-free system (WGCFS; CellFree Sciences, Matsuyama, Japan) to synthesize recombinant PfPV1. The expressed recombinant protein was Ni^2+^ affinity purified with an expected band at ~60 kDa (Fig. 1b). The purified recombinant PfPV1 protein was subsequently used to raise antibodies in rabbit. The antibodies specifically recognized blood-stage parasites native PfPV1 by Western blot, in both reducing and non-reducing conditions (Fig. 1c). Recognition of native PfPV1 under reducing conditions was at a slightly higher molecular weight.

### Subcellular localization of PfPV1 in comparison with EXP2

**Figure 2:**
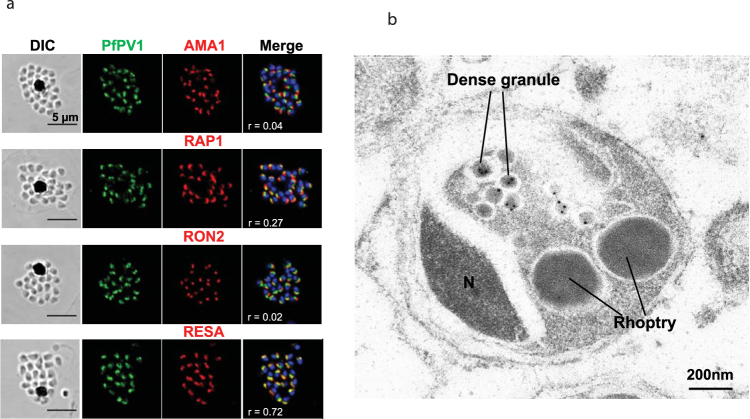
Subcellular localization of PfPV1 in schizont-stage parasites. (**a**) Results of IFA with Confocal Microscopy. Labeling of the utilized antibodies is on the upside of panels. PfPV1 (green) was counter stained with four different markers of subcellular localizations (red). The parasite nucleus was stained with DAPI (blue). PfPV1 co-localized with RESA a dense granule marker. DIC; Differential interference contrast microscope. Merge; the image created by merging image of IFA, and nuclear-staining. Pearson’s coefficient values ( = r) were calculated by using Zen 2010 software (Carl Zeiss MicroImaging, Thornwood, NY) and shown on the merged panels. Scale bar = 5 µm. (**b**) Results of IEM using rabbit anti-PfPV1 antibody. Gold particles can be observed at the *Plasmodium falciparum* merozoite dense granules at schizont stage. N; nuclear. Scale bar = 200 nm.

Using the rabbit anti-PfPV1 antibodies, we conducted subcellular localization of PfPV1 in schizont stage parasites by immunofluorescence assay (IFA) (Fig. 2a). We determined the co-localization intensity correlation by Pearson’s correlation coefficients (r) between PfPV1 and different organelle markers. PfPV1 showed tighter co-localization with RESA (r = 0.72), a dense granule protein, than with AMA1 (r = 0.04), RAP1 (r = 0.27) and RON2 (r = 0.02), which are microneme, rhoptry body and rhoptry neck proteins respectively. These results suggested that PfPV1 is a *P*. *falciparum* merozoite dense granule protein. Immunoelectronmicroscopy (IEM) examination confirmed the localization of PfPV1 to the dense granules (Fig. 2b). Moreover, consistent with a previous report indicating GFP tagged PfPV1 localizes to PV^11^, the IFA of ring and trophozoite stage parasites showed that PfPV1 co-localized with RAP1 a PV protein, but not with RESA or SBP1, which are erythrocyte membrane and Maurer’s clefts proteins respectively (Figure S1).

**Figure 3:**
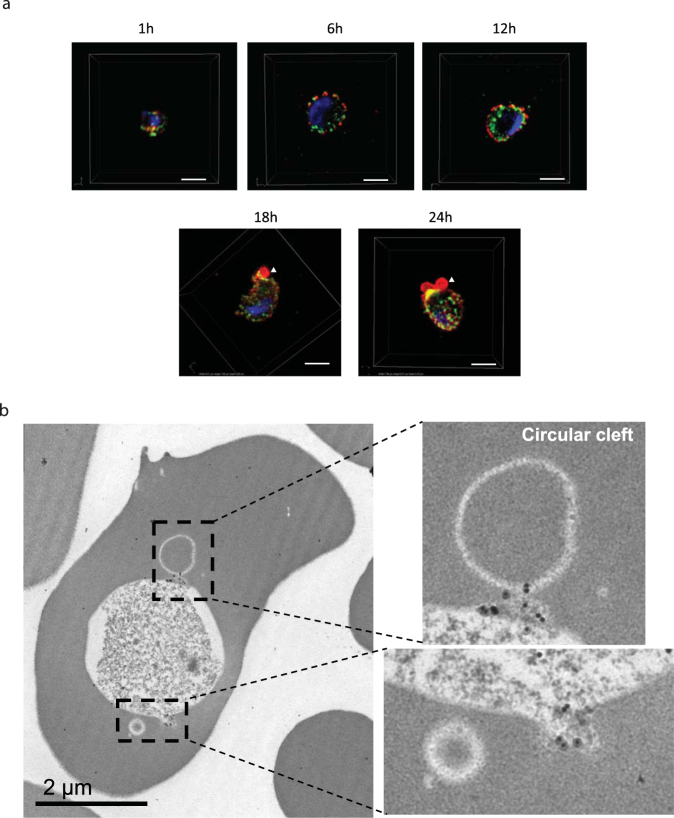
Subcellular localization of PfPV1 in ring- and trophozoite-stage parasites. (**a**) Results of IFA with SIM. Samples were prepared shortly after merozoite invasion as indicated. PfPV1 (green) was counter stained with EXP2 (red). Parasite nucleus was stained with DAPI (blue). Arrowheads indicate observed region of PV protrusions with accumulated EXP2. The images were made from stacks of SIM optical sections (120 nm Z-step size). Scale bars = 2 µm. (**b**) Results of IEM using rabbit anti-PfPV1 antibody. Gold particle labeling was observed at the base of circular clefts. Scale bar = 2 µm.

With an understanding that a previous report demonstrated that PTEX components localize to the merozoite dense granules^8^, we hypothesized that PfPV1 could play an important role in protein export to the erythrocyte cytosol after invasion. We therefore utilized structured illumination microscopy (SIM) to comparatively examine the sub-cellular localization of PfPV1 and EXP2. Consistent with previous reports^27^, EXP2 was observed in PV 1–12 h in early and mid trophozoite stages (Fig. 3a), while 18–24 h post invasion, it accumulated at a PV protrusion (Fig. 3a, arrowheads). Similar to other PTEX associated proteins^27^, PfPV1 localized proximal to EXP2, and remained partially co-localized in the time course, albeit at varying degree of co-localization (Fig. 3a). In addition, PfPV1 also accumulated at the base of the PV protrusion in partial co-localization with EXP2 18–24 h after invasion (Fig. 3a; yellow signals). IEM examination of trophozoite stage parasites with the rabbit anti-PfPV1 showed accumulated gold particles at the base of circular cleft or tubular structure (Fig. 3b, insets), hence confirming the SIM findings (Fig. 3a, 18–24 h).

### Identification of PfPV1 associated proteins

**Figure 4:**
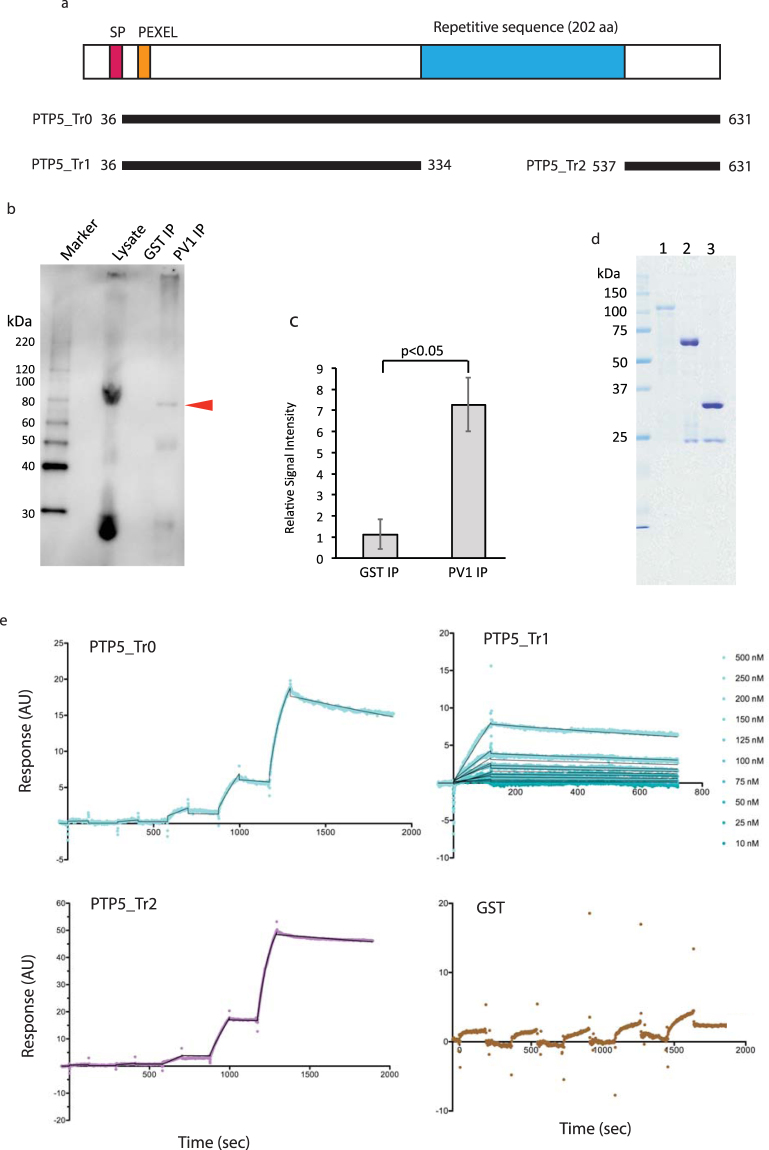
Analysis of recombinant proteins; PTP5 and PfPV1 interaction. (**a**) Schematic presentation of PTP5 protein. SP (Red) is the predicted signal peptide, PEXEL (Orange) is the PEXEL motif position, the repetitive sequence is in Blue, Thick black lines indicate truncation design of PTP5 proteins expressed using WGCFS. (**b**) Results of immunoprecipitation experiment using rabbit anti-PfPV1 antibody. For Western blot analysis, we used mouse anti-PTP5 antibody to detect PTP5. Marker; protein molecular weight marker, Lysate; parasite lysate derived from 10^8^ trophozoite-/schizont-rich parasite pellet, GST IP; sample immunoprecipitated with rabbit anti-GST antibody and included as Negative control, PV1 IP; sample immunoprecipitated with anti-PfPV1 antibody. The arrowhead indicates a signal that corresponds to native PTP5. Three independent experiments were carried out (Figure S2). (**c**) Relative intensities derived from PTP5 on the Western blot analysis are presented as a bar graph. The background signal was defined as the signal in the empty lane that corresponded to the molecular weight of native PTP5 (approximately 80 kDa). Error bars represent SD. Significantly higher signals from PTP5 were detected from the sample immunoprecipitated with anti-PV1 antibody (p < 0.05). (**d**) Purified GST-tagged recombinant PTP5 resolved in SDS-PAGE and stained with CBB. Lane 1; PTP5_Tr0, Lane 2; PTP5_Tr1, Lane 3; PTP5_Tr2. (**e**) SPR kinetics analyses sensorgrams. His-tagged purified recombinant PfPV1 was used for ligand. Analytes used for the analyses were indicated in sensorgrams. Black lines indicate fits used for the calculation of kinetics parameters. The PTP5_Tr0 concentrations used for the single-cycle kinetic analysis; 0.16, 0.8, 4, 20 and 100 nM, The PTP5_Tr1 concentrations used for the multi-cycle kinetic analysis; 10, 25, 50, 75, 100, 125, 150, 200, 250 and 500 nM, The PTP5_Tr2 concentrations used for the single-cycle kinetic analysis; 0.24, 1.2, 6, 30, and 150 nM. The GST concentrations used for the single-cycle kinetic analysis; 0.96, 4.8, 24, 120, 600 nM.

To investigate the role of PfPV1 in protein export, we explored for possible interacting proteins. We conducted immunoprecipitation experiments with rabbit anti-PfPV1 antibody on Nonidet P-40 solubilized parasites samples. We then carried out Western blot analysis on the immunoprecipitate with a panel of mouse antibodies raised against 286 *P*. *falciparum* recombinant proteins (Table S2). PfEMP1-trafficking protein (PTP5, PF3D7_1002100)^16,17^ was co-immunoprecipitated with PfPV1 (Fig. 4b arrowhead, and S2a). The signal intensity derived from PTP5 was significantly higher than that of samples co-immunoprecipitated with anti-GST antibody (Fig. 4c). Inversely, rabbit anti-PTP5 antibody co-immunoprecipitated PV1 with PTP5 (Figure S2b). PTP5 consists of 631 amino acids (aa), with a theoretical MW of 69.9 kDa. The protein has a signal peptide (Fig. 4a, SP) and a PEXEL motif (RLLSE) starting at R_57_ (Fig. 4a, PEXEL). In addition, PF3D7_0801000, one of the proteins exported through PTEX^5,8^, was also co-immunoprecipitated with PfPV1 (Figure S3). EXP2 and HSP101 were also co-immunoprecipitated (Figure S4), in line with previous studies^12,13^. In contrast, RAP1, which localizes to PV space, was not co-immunoprecipiated with PV1 (Figure S2c).

### PfPV1 and PTP5 protein-protein interaction

We utilized surface plasmon resonance (SPR) to demonstrate and characterize direct protein-protein interaction between PfPV1 and PTP5. Kinetics analysis of the WGCFS expressed PfPV1 and PTP5_Tr0; 36 aa – 631 aa (Figs 1b and 4a,d) showed direct interaction with a *K*_D_ value of 2.705 × 10^−9^ M in 1:1 stoichiometry (Fig. 4e). To identify the PTP5 region that is essential for interaction with PfPV1, we truncated PTP5 into PTP5_Tr1; 36 aa – 334 aa, and PTP5_Tr2; 537 aa – 631 aa (Fig. 4a,d). The interaction between PTP5_Tr2 and PfPV1 exhibited a *K*_D_ value of 1.236 × 10^−9^ M, while as that of PTP5_Tr1 and PfPV1 showed a *K*_D_ value of 9.381 × 10^−7^M (Fig. 4e). The PTP5_Tr1-PfPV1 interaction was 759 fold weaker than that of PTP5_Tr2-PfPV1, which prompted determination of interaction kinetics parameters through multi-cycle kinetic analyses (Fig. 4e). The results indicated that, at least, PTP5 region 335 aa − 631 aa is essential for PfPV1 high affinity interaction, and thus hereinafter referred to as PfPV1 High-affinity Region (PHR). Similarly, we also observed a direct interaction between PfPV1 and PF3D7_0801000 (Figure S3).

### Subcellular localization of PTP5FL-HA and PTP5ΔPHR-HA

**Figure 5:**
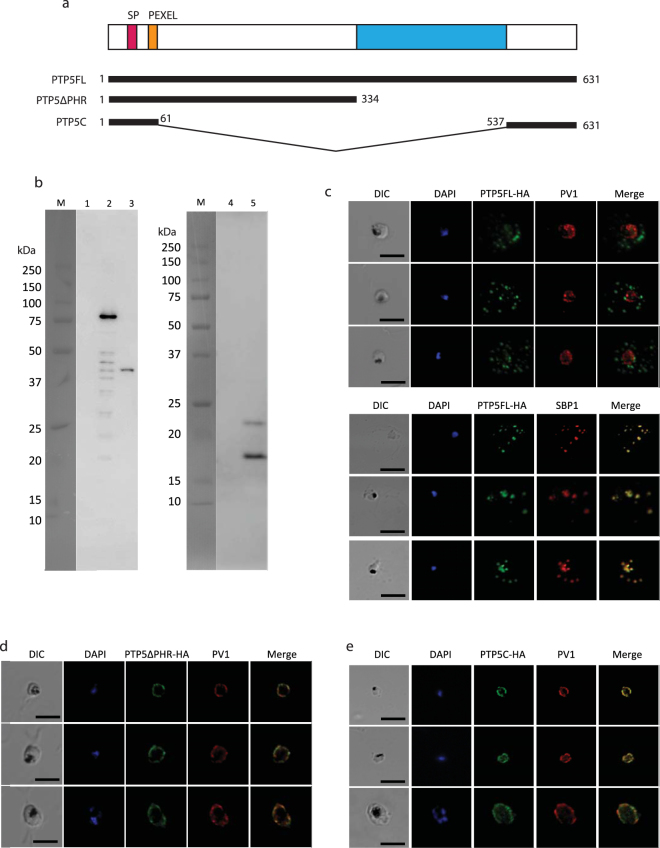
Characterization of episomally expressed PTP5, PTP5ΔPHR and PTP5C. (**a**) Schematic presentation of PTP5 protein episomally expressed in *P*. *falciparum* 3D7 strain. The color code is same as Fig. 4a. (**b**) Western blot analysis of parasite lysate expressing PTP5FL-HA, PTP5ΔPHR-HA and PTP5C-HA. Rat anti-HA antibody was used for the primary antibody. M; All Blue prestained protein molecular weight marker, 1; 3D7 parasite lysate (negative control, prepared from 1 × 10^6^ infected RBC), 2; PTP5FL-HA parasite lysate (prepared from 1 × 10^6^ infected RBC), 3; PTP5ΔPHR-HA parasite lysate (prepared from 1 × 10^6^ infected RBC), 4; 3D7 parasite lysate (negative control, prepared from 1 × 10^7^ infected RBC), 5; PTP5C parasite lysate (prepared from 1 × 10^7^ infected RBC). (**c**,**d**,**e**) Subcellular localization of PTP5FL-HA, PTP5ΔPHR-HA and PTP5C-HA. The samples were stained with anti-HA (green) co-stained with anti-PfPV1 or anti-SBP1 (red). Parasite nucleus was stained with DAPI (blue). Scale bars = 5 µm.

**Figure 6:**
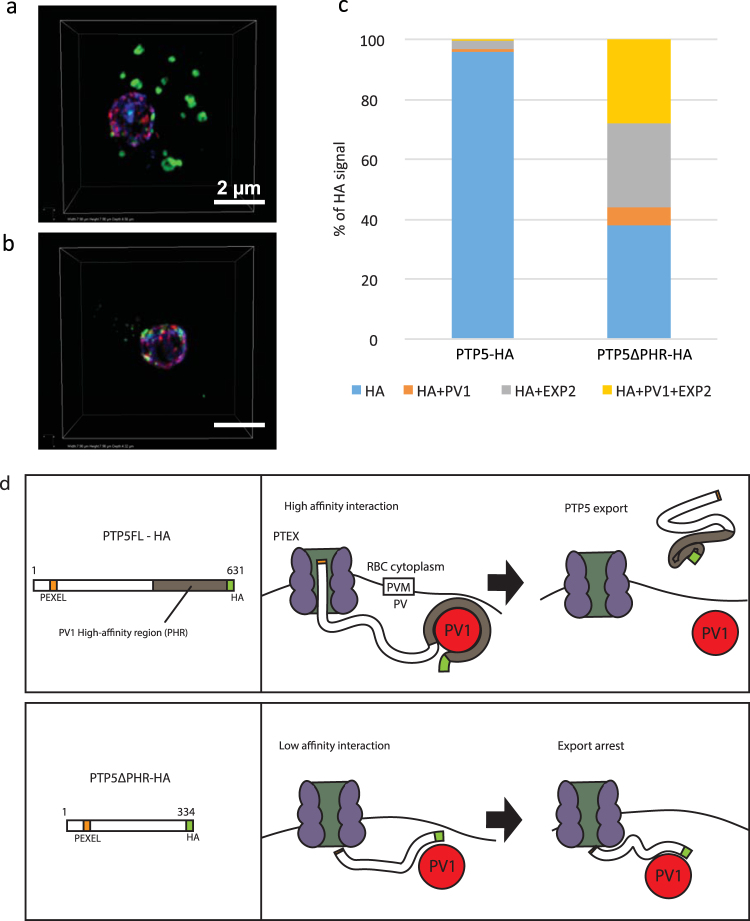
3D-SIM observations of episomally expressed PTP5 and PTP5ΔPHR. (**a**) Subcellular localization of PTP5FL-HA (green) and (**b**) PTP5ΔPHR-HA (green) observed with SIM. EXP2 (red) and PfPV1 (cyan) were used as a counter-staining. The results of 3D-SIM for the identical erythrocytes are available in Movies S1 and S2. (**c**) Numerical analysis of protein localizations; vertical axis shows percentages of HA signals detected in 3D-SIM. HA; HA signals did not co-localized with other proteins, HA + PV1; HA signals co-localized with PV1 signals, HA + EXP2; HA signals co-localized with EXP2 signals, HA + PV1 + EXP2; HA signals co-localized with the other 2 proteins. The HA signal percentages are average values of 3 PTP5FL-HA, and 5 PTP5ΔPHR-HA observed parasite cells. (**d**) Schematic presentation of PTP5FL-HA (upper panel) and PTP5ΔPHR-HA (lower panel). PEXEL motif, PV1 high-affinity region (PHR), and HA tag of episomally expressed PTP5 are represented in orange, gray, and green colors respectively. The present study identified PHR as essential for PTP5 export, suggesting that PV1 plays an important role in protein translocation mediated by the PTEX complex. The PTP5FL-HA that interacted with PV1 successfully translocated to the cytosol of infected erythrocyte. PTP5ΔPHR-HA mutant weakly interacts PV1 and the export of this protein was arrested at PV.

To analyze whether the PfPV1-PTP5 interaction contributes to PTP5 export, we generated transgenic parasite lines episomally expressing either PTP5FL (1 aa – 631 aa), PTP5ΔPHR (1 aa – 334 aa) or PTP5C (1 aa – 61 aa fused to 537 aa – 631 aa) followed by 3 × HA at the C-terminus (Fig. 5a). We confirmed expression of the proteins by Western blot and IFA using anti-HA antibodies. Notably, PTP5FL-HA, truncates PTP5ΔPHR-HA and PTP5C-HA were detected by immunoblot at the expected molecular weights (Fig. 5b). We however also observed an extra band with PTP5C-HA probably arising from a non-processed episomally expressed protein (Fig. 5b). Moreover, analysis with IFA demonstrated patchy signals of PTP5FL-HA in the cytosol of the infected erythrocyte existing in co-localization with SBP1; a Maurer’s cleft marker (Fig. 5c), suggesting that this PTP5 is exported to Maurer’s clefts. However, PTP5ΔPHR-HA and PTP5C-HA were not exported but arrested in the PV space and co-localized with PfPV1 (Fig. 5d,e). We therefore, conducted additional localization experiments using 3D-SIM (Fig. 6a,b and Movies S1, S2), to explore the loci of PTP5ΔPHR-HA accumulation in the PV space. We observed that only 1% of PTP5FL-HA co-localized with both PV1 and EXP2, while 30% of PTP5ΔPHR-HA had co-localization with both of the two PV proteins (Fig. 6c). Taken together, these results suggested that PTP5ΔPHR-HA translocation was arrested at an intermediate complex with PfPV1 and PTEX.

In order to confirm the protein-protein interaction between PV1 and PTP5FL-HA or PTP5ΔPHR-HA in the parasite, we performed immunoprecipitation of the transfected parasites using anti-PfPV1 antibody. Consistent with the SPR findings, both PTP5FL-HA and PTP5ΔPHR-HA were co-immunoprecipitated by anti-PV1 antibody (Figure S5a). In addition, PV1 was co-immunoprecipitated with PTP5FL-HA or PTP5ΔPHR-HA by anti-HA antibody (Figure S5b).

## Discussion

*Plasmodium falciparum* merozoite dense granules release their contents into PV space soon after erythrocyte invasion^28^. For instance, RESA, a protein stored in dense granules, is exported to the infected erythrocyte cytosol within 12 min post invasion^27^. Thus, it is hypothesized that proteins localized to the dense granules play a role in protein export into infected erythrocytes for hastened construction of export machinery. Therefore, there is need to screen and characterize novel dense granule proteins that could be involved in protein export. In this study, using IEM we demonstrated PfPV1 as a novel *P*. *falciparum* merozoite dense granule protein, and a *bona fide* PV protein as previously suggested^11^.

It’s recently reported that PfPV1 co-precipitated with PTEX complex^12,13^ suggesting this protein constitutes PTEX accessory molecules^13^. Consistently, our results here showed that PfPV1 is partially co-localized with EXP2 (Figs 3a and 6). We demonstrated that PV1 directly interacted with PTP5, thus an intermediate complex as PTEX-PTP5-PfPV1 is conjectured (Fig. 6d). The observed high affinity of PfPV1 and PTP5, suggests that the PfPV1-PTP5 is a “strong transient complex”^29^. Thus, it can be postulated that a structural change of the PfPV1-PTP5 complex caused by an unknown protein, and/or PTP5 unfolding mediated by PTEX would be required to dissociate PTP5 for export.

This study reports, for the first time, the biochemical properties of protein-protein interactions between PV1, PTP5 and PF3D7_0801000. To the best of our knowledge, this is also the first study reporting direct interaction between PTEX accessory proteins and exported proteins. PTP5_Tr0 and PTP5_Tr2 bound PfPV1 with comparable *K*_D_ values of 2.705 × 10^−9^ M, and 1.236 × 10^−9^ M, respectively. We therefore, considered PTP5_Tr2 as a principal PTP5 region attributable to PfPV1 binding. We also demonstrated that PfPV1 and PF3D7_0801000 interact directly. However, we could not identify any shared amino acid sequence/motif(s) between PTP5_Tr2, and PF3D7_0801000 that would be responsible for the PfPV1 binding. These findings suggest two possibilities regarding the interaction of these molecules. Either, the interaction is probably dependent on multiple structural motifs or, the binding motif lacks conserved sequence, but is dependent on amino acid hydrophobicity and sequence length are contributory to binding, as observed in signal peptides^30^. Further biochemical analysis, in future, would elucidate the mechanism of interaction and identify the specific PfPV1 binding motifs.

The importance of PfPV1-PTP5 interaction to PTP5 export was explored through a comparative subcellular localization of PTP5FL-HA, PTP5ΔPHR-HA and PTP5C-HA. We demonstrated that PTP5ΔPHR-HA export was arrested in PV space (Figs 5d and 6b), suggesting a critical role of PHR in the PTP5 export. This observation highlights, for the first time, the essential role of a protein’s C-terminal region in its export. The PTP5ΔPHR-HA arrested in export has an intact N-terminal sequence consisting of native PEXEL and PEXEL-proximal downstream residues. Previous reports indicate that the less conserved residues downstream of matured N-terminus are considered responsible for protein export^31,32^. Furthermore and consistent with this observation, PTP5C-HA export was similarly arrested in the PV space (Fig. 5e). Hence, it was proposed that both the downstream PEXEL and PHR are essential for PTP5 export.

The exported proteins are proteolytically cleaved by PMV on the C-terminal side of the conserved leucine in the PEXEL sequence RxLxE/D/Q and is N-acetylated (Ac-xE/Q/D)^20^. After PMV cleavage, further steps in export such as the mechanism of protein recognition at PV space, remains unclear. Our findings suggest that PTEX auxiliary proteins such as PfPV1 play an important role in export protein recognition in the PV space. However, further in-depth biochemical analysis is required to elucidate PfPV1 – protein interactions required for its role in the export protein recognition.

Recently, while this paper was being reviewed, Steven, B. *et al*. reported on the findings of PV1-HA interacting proteins mass spectrometry as well as its knock down phenotype analyses^33^. Consistent with our results, it was reported that PV1-HA co-precipitated some exported proteins and PTEX components, as well as co-localized with dense granule protein when observed by IFA. Notably, knockdown of PV1 resulted in altered knob morphology, reduced cell rigidity, and significant decrease in expression of export proteins such as PfEMP1, KAHRP, and PfEMP3. This is also in line with our hypothesis that PV1 is important for protein export. Further study is needed to clarify the exact role of interaction between PV1 and PTP5 for subsequent PTP5 export.

In conclusion, PfPV1 was characterized as a novel merozoite dense granule protein that interacts with proteins exported to the cytosol of an infected erythrocyte. Our biochemical analyses demonstrated that PfPV1 interacts directly with PTP5 via PTP5 PHR; a region identified in this study. Moreover, the arrest of PTP5ΔPHR-HA export at the PV strongly suggests that PHR is essential for PTP5 export. Put together, the PfPV1 data presented here strengthens the need for further studies aimed at unraveling mechanisms of protein export crucial for parasite growth and virulence, which could offer novel approaches to malaria intervention.

## Materials and Methods

### Construction of plasmids and preparation of purified recombinant proteins

All the sequences of primers for the construction of plasmids used in this study are summarized in Table S1. *Pfpv1* and *ptp5_tr0* were amplified from cDNA derived from *P*. *falciparum* 3D7 strain using primers; PV1F and PV1R, PTP5F and PTP5R respectively. *Ptp5_tr1* and *ptp5_tr2* were amplified from plasmids harboring *pfptp5_tr0* using primers; PTP5F and PTP5Tr1R, PTP5Tr2F and PTP5R respectively. N-terminal truncate of Pf3D7_0801000 (*Pf3D7_0801000N*) spanning 93 aa – 494 aa was amplified from cDNA derived from *P*. *falciparum* 3D7 strain using primers; 0801NF and 0801NR. The amplified DNA was restricted with XhoI and NotI, and ligated into WGCFS vector, pEU, for N-terminal glutathione S-transferase (GST) or C-terminal hexa-histidine (His) fusion protein expression (CellFree Sciences, Matsuyama, Japan). *Ptp5fl* was PCR amplified from *P*. *falciparum* 3D7 strain cDNA using primers PTP5FL-F and PTP5FL-R. *Ptp5Δphr* was amplified from plasmids harboring *ptp5fl* using primers PTP5FL-F and PTP5ΔPHR-R. *Ptp5c* gene was commercially synthesized by GenScript (Tokyo, Japan). The resulting DNA fragments were restricted with NotI and PstI, and ligated into pD3HA plasmids for episomal expression of PTP5FL, PTP5ΔPHR and PTP5C^34^.

### Production of mouse and rabbit antisera

As previously described^35^, we generated rabbit polyclonal antisera against PfPV1 (V_23_ to S_452_ 3D7 sequence; PF3D7_1129100) and mouse antisera against PTP5 (T_36_ to K_631_; PF3D7_ PF3D7_1002100), AMA1 (Q_25_ to K_546_; PF3D7_1133400), RAP1 (M_1_ to D_782_; PF3D7_1410400), EXP2 (D_25_ to E_287_; PF3D7_1471100), SBP1 (Q_258_ to T_337_; PF3D7_0501300) and RON2 (K_84_ to Q_968_; PF3D7_1452000). All antigens were synthesized by WGCFS with N or C-terminal His- or GST-tag fusion protein expression. All animal antisera were purchased from KITAYAMA LABES Co., LTD. (Ina, Japan). Mouse monoclonal antibody against RESA (PF3D7_0102200) was a kind gift from Robin F. Anders^36^.

### Western blot analysis

Western blot was conducted as previously described^37^. Purified schizont-rich parasite pellets from *P*. *falciparum* 3D7 strain were directly lysed in reducing or non-reducing SDS-PAGE sample buffer. The lysate was boiled at 95 °C for 5 min, centrifuged at 10,000 × g for 10 min at 4 °C, supernatant collected, and resolved by SDS-PAGE on a 12.5% e-PAGEL (ATTO). Following electroblotting, a polyvinylidene fluoride membrane was incubated with primary antibodies diluted at the following concentrations: rabbit anti-PfPV1 antibody, 1:1000; mouse anti-PTP5 antibody, 1:1000; rat anti-HA monoclonal antibody (Roche Diagnostics, Indianapolis, IN, USA), 1:100; mouse anti-PF3D7_0801000 antibody, 1:1000. After washing, the membrane was incubated with secondary antibodies diluted at the following concentrations: ECL peroxidase labeled anti-mouse antibody (GE Healthcare), 1:10000; ECL peroxidase labeled anti-rabbit antibody (GE Healthcare), 1:10,000; ECL peroxidase labeled anti-rat antibody (GE Healthcare), 1:1000.

### Immunofluorescence Assay

IFA was conducted as previously described^35^. Briefly, samples were incubated with primary antibodies diluted at the following concentrations in blocking solution at 37 °C for 1 h. Rabbit anti-PfPV1 antibody, 1:1000; mouse anti-AMA1 antibody, 1:100; mouse anti-RAP1 antibody, 1:1,000; mouse anti-RON2 antibody, 1:100; mouse anti-RESA monoclonal antibody, 1:100; mouse anti-EXP2 antibody, 1:1000; mouse anti-SBP1 antibody, 1:100; rat anti-HA antibody, 1:100. Secondary antibodies, Alexa Fluor 488-conjugated goat anti-rabbit IgG, Alexa Fluor 488-conjugated goat anti-rat IgG, Alexa Fluor 568-conjugated goat anti-rabbit IgG, Alexa Fluor 568-conjugated goat anti-mouse IgG and Alexa Fluor 647-conjugated goat anti-mouse IgG (Invitrogen), were used at a 1:1000 dilution in blocking solution at 37 °C for 30 min. DAPI (4′, 6-diamidino-2-phenylindole) at 2 µg/ml was also added to the secondary-antibody solution to stain the nuclei. Slides were mounted in ProLong Gold Antifade reagent (Invitrogen) and observed under a 63 × oil immersion lens. Image capture and processing were performed with a confocal scanning laser microscope (LSM710; Carl Zeiss MicroImaging, Thornwood, NY). Images were processed in Adobe Photoshop (Adobe Systems Inc., San Jose, CA).

### Structured illumination microscopy (SIM) imaging and image processing

The samples were prepared is same manner as for IFA. Nikon N-SIM system (Nikon, Tokyo, Japan) equipped with a 100×/1.49 TIRF oil immersion objective lens (Nikon), the iXon + electron multiplying charged-coupled device camera (Andor) and an excitation laser unit of 405 nm, 488 nm, 561 nm and 640 nm (Coherent) was used for a super-resolution optical imaging. Z-stacks of SIM optical sections were acquired with a 120 nm Z-step size. Image processing, including 3-dimensional reconstruction and co-localization analysis, were carried out using the NIS-Element Advanced Research software (Nikon).

### Immunoprecipitation and detection

Immunoprecipitation was carried out as described^38^. Briefly, proteins were extracted from late trophozoites-/schizont-rich parasite pellets in PBS with 0.5% Nonidet P-40 containing cOmplete^TM^ protease inhibitor (Roche). Seventy-five μl of the supernatants were preincubated at 4 °C in suspension of protein G-conjugated beads (protein G-Sepharose 4 Fast Flow; GE Healthcare) in TNE buffer (50 mM Tris-HCl, 0.2 M NaCl, 5 mM EDTA). Aliquots of recovered supernatants were incubated with 3 μl of rabbit anti-PfPV1 or anti-GST antibodies. The recovered supernatant aliquots were again mixed with the suspension of protein G-conjugated bead. After 2 h incubation at 4 °C with continuous rotation, the beads were washed three times with TNE buffer. Finally, proteins were extracted from the protein G-conjugated beads by incubation with 1 × SDS-PAGE reducing loading buffer at 95 °C for 5 min. Final supernatants were used for Western blot analysis using panel of mouse antisera against 286 antigens synthesized by WGCFS (Table S2) at the same dilution (1/1000). When the immunoprecipitated samples were screened, we applied the following criteria for the selection; 1) single major band in the anti-PV1 imunoprecipitated sample should be detected; 2) the band should not be detected in negative control sample (immunoprecipitation sample prepared with anti-GST antibody). Only the two proteins among the 286 passed the criteria. The positive signal of the co-immunprecipitated PTP5 (n = 3) was quantified by Image-J (https://imagej.nih.gov/ij/). The relative density was determined in relation to the background in empty lane (Fig. 4c).

### Surface Plasmon Resonance

All SPR experiments were performed using a Biacore X100 instrument (GE Healthcare) according to manufacturer’s instructions. The Biacore X100 evaluation software was used for single-cycle or multi-cycle kinetic analysis. Sensor CM5, amine coupling reagents, and buffers were purchased from GE Healthcare. Fresh HBS-EP + (10 mM HEPES, pH 7.4, 150 mM NaCl, 3 mM EDTA, 0.05% (v/v) surfactant P20) was used as running buffer for all SPR experiments. Blank flow cells were used to subtract buffer effects on sensorgrams. After subtraction of the contribution of bulk refractive index, and nonspecific interactions with the CM5 chip surface, individual association (*k*_a_) and dissociation (*k*_d_) rate constants were obtained by global fitting of data. Measurement conditions were optimized so that the contribution of mass transport to the observed values of *K*_D_ was negligible.

### Immunoelectron microscopy

Parasites were fixed, embedded in LR White resin (Polysciences, Warrington, PA), and ultrathin sections were immunostained as previously described^37^. Rabbit anti-PfPV1 antibody was used at a dilution of 1:200. Samples were examined with transmission electron microscope (JEM-1230; JEOL, Tokyo, Japan).

### *P*. *falciparum* culture and transfection

*P*. *falciparum* 3D7 strain was a kind gift from the National Institute of Allergy and Infectious Diseases (NIAID), and cultured as previously described^39^. Briefly, parasites were maintained in continuous culture of 2% hematocrit O^+^ human erythrocytes, incubated at 37 °C in RPMI-1640 complete medium. RPMI-1640 complete medium was supplemented with 5% heat-inactivated pooled type AB^+^ human serum and 0.25% Albumax II (Invitrogen, Carlsbad, CA), 200 mM hypoxanthine (Sigma, St. Louis, MO) and 10 µg/ml gentamicin (Invitrogen). The human erythrocytes and plasma were obtained from the Japanese Red Cross Society. For transfection, parasitized erythrocytes were resuspended in 200 µl of cytomix (120 mM KCl, 0.15 mM CaCl_2_, 2 mM EGTA, 5 mM MgCl_2_, 10 mM K_2_HPO_4_/KH_2_PO_4_, 25 mM HEPES, pH 7.4) containing 100 µg of plasmid DNA. Electroporation was performed in 2 mm cuvette using a Gene Pulser Xcell Electroporation System (Bio-Rad, Hercules, CA) at the following conditions; 0.31 kV, 950 µF and ∞ Ω. After electroporation, the erythrocytes were immediately resuspended in complete medium. WR99210 was added to 10 nM in the culture medium 1 day after electroporation, and culture was maintained under same drug pressure until drug-resistant parasites appeared.

### Ethical approval

The use of human erythrocytes and human plasma for parasite culture was approved by the ethical review committee of Ehime University Hospital (Aidaiibyourin 1301005). The experiment was conducted in accordance with approved protocols and regulations.

## Electronic supplementary material

Supplementary Information

Supplementary Table S2

Movie S1

Movie S2

## References

[1] WHO. World Malria Report2016 (2016).

[2] Spillman NJ, Beck JR, Goldberg DE (2015). Protein export into malaria parasite-infected erythrocytes: mechanisms and functional consequences. Annu Rev Biochem.

[3] Raventos-Suarez C, Kaul DK, Macaluso F, Nagel RL (1985). Membrane knobs are required for the microcirculatory obstruction induced by *Plasmodium falciparum*-infected erythrocytes. Proceedings of the National Academy of Sciences.

[4] Heiber A (2013). Identification of new PNEPs indicates a substantial non-PEXEL exportome and underpins common features in *Plasmodium falciparum* protein export. PLoS Pathog.

[5] de Koning-Ward TF (2009). A newly discovered protein export machine in malaria parasites. Nature.

[6] Beck JR, Muralidharan V, Oksman A, Goldberg DE (2014). PTEX component HSP101 mediates export of diverse malaria effectors into host erythrocytes. Nature.

[7] Elsworth B (2014). PTEX is an essential nexus for protein export in malaria parasites. Nature.

[8] Bullen HE (2012). Biosynthesis, localization, and macromolecular arrangement of the *Plasmodium falciparum* translocon of exported proteins (PTEX). Journal of Biological Chemistry.

[9] Matthews K (2013). The Plasmodium translocon of exported proteins (PTEX) component thioredoxin‐2 is important for maintaining normal blood‐stage growth. Molecular microbiology.

[10] Matz JM, Matuschewski K, Kooij TW (2013). Two putative protein export regulators promote Plasmodium blood stage development *in vivo*. Molecular and biochemical parasitology.

[11] Chu T, Lingelbach K, Przyborski JM (2011). Genetic evidence strongly support an essential role for PfPV1 in intra-erythrocytic growth of P. falciparum. PloS one.

[12] Mesén-Ramírez P (2016). Stable Translocation Intermediates Jam Global Protein Export in Plasmodium falciparum Parasites and Link the PTEX Component EXP2 with Translocation Activity. PLoS Pathog.

[13] Elsworth B (2016). Proteomic analysis reveals novel proteins associated with the Plasmodium protein exporter PTEX and a loss of complex stability upon truncation of the core PTEX component, PTEX150. Cellular microbiology.

[14] Leech JH, Barnwell JW, Miller LH, Howard RJ (1984). Identification of a strain-specific malarial antigen exposed on the surface of Plasmodium falciparum-infected erythrocytes. The Journal of experimental medicine.

[15] Su XZ (1995). The large diverse gene family var encodes proteins involved in cytoadherence and antigenic variation of *Plasmodium falciparum*-infected erythrocytes. Cell.

[16] Maier AG (2008). Exported proteins required for virulence and rigidity of *Plasmodium falciparum*-infected human erythrocytes. Cell.

[17] Tshefu K, James M (1995). Relationship of antibodies to soluble *Plasmodium falciparum* antigen (Pf70) and protection against malaria in a human population living under intense transmission in Kinshasa, Zaire. Tropical medicine and parasitology: official organ of Deutsche Tropenmedizinische Gesellschaft and of Deutsche Gesellschaft fur Technische Zusammenarbeit (GTZ).

[18] Hiller NL (2004). A host-targeting signal in virulence proteins reveals a secretome in malarial infection. Science.

[19] Marti M, Good RT, Rug M, Knuepfer E, Cowman AF (2004). Targeting malaria virulence and remodeling proteins to the host erythrocyte. Science.

[20] Boddey JA, Moritz RL, Simpson RJ, Cowman AF (2009). Role of the Plasmodium export element in trafficking parasite proteins to the infected erythrocyte. Traffic.

[21] Boddey JA (2013). Role of plasmepsin V in export of diverse protein families from the *Plasmodium falciparum* exportome. Traffic.

[22] Dixon MW (2008). Targeting of the Ring Exported Protein 1 to the Maurer’s Clefts is Mediated by a Two‐Phase Process. Traffic.

[23] Haase S (2009). Sequence requirements for the export of the *Plasmodium falciparum* Maurer’s clefts protein REX2. Molecular microbiology.

[24] Blisnick T (2000). Pfsbp1, a Maurer’s cleft *Plasmodium falciparum* protein, is associated with the erythrocyte skeleton. Molecular and biochemical parasitology.

[25] Aikawa M (1990). Pf155/RESA antigen is localized in dense granules of *Plasmodium falciparum* merozoites. Experimental parasitology.

[26] Nyalwidhe J, Lingelbach K (2006). Proteases and chaperones are the most abundant proteins in the parasitophorous vacuole of *Plasmodium falciparum*‐infected erythrocytes. Proteomics.

[27] Riglar DT (2013). Spatial association with PTEX complexes defines regions for effector export into *Plasmodium falciparum*-infected erythrocytes. Nature communications.

[28] Torii M, Adams JH, Miller LH, Aikawa M (1989). Release of merozoite dense granules during erythrocyte invasion by *Plasmodium knowlesi*. Infection and immunity.

[29] Perkins JR, Diboun I, Dessailly BH, Lees JG, Orengo C (2010). Transient protein-protein interactions: structural, functional, and network properties. Structure.

[30] Chou MM, Kendall DA (1990). Polymeric sequences reveal a functional interrelationship between hydrophobicity and length of signal peptides. Journal of Biological Chemistry.

[31] Boddey JA (2016). Export of malaria proteins requires co-translational processing of the PEXEL motif independent of phosphatidylinositol-3-phosphate binding. Nature communications.

[32] Grüring C (2012). Uncovering common principles in protein export of malaria parasites. Cell host & microbe.

[33] Steven B (2017). An exported protein-interacting complex involved in the trafficking of virulence determinants in *Plasmodium*-infected erythrocytes. Nature communications.

[34] Riglar DT (2011). Super-resolution dissection of coordinated events during malaria parasite invasion of the human erythrocyte. Cell host & microbe.

[35] Ito D (2013). RALP1 is a rhoptry neck erythrocyte-binding protein of Plasmodium falciparum merozoites and a potential blood-stage vaccine candidate antigen. Infect Immun.

[36] Culvenor JG, Day KP, Anders RF (1991). *Plasmodium falciparum* ring-infected erythrocyte surface antigen is released from merozoite dense granules after erythrocyte invasion. Infect Immun.

[37] Arumugam TU (2011). Discovery of GAMA, a *Plasmodium falciparum* merozoite micronemal protein, as a novel blood-stage vaccine candidate antigen. Infect Immun.

[38] Ito D (2011). Plasmodial ortholog of *Toxoplasma gondii* rhoptry neck protein 3 is localized to the rhoptry body. Parasitology international.

[39] Trager W, Jensen JB (1976). Human malaria parasites in continuous culture. Science.

