# Supplementary material for: PV1, a novel Plasmodium falciparum merozoite dense granule protein, interacts with exported protein in infected erythrocytes
Source: Sci Rep. 2018 Feb 27;8:3696. doi: 10.1038/s41598-018-22026-0 (PMC5829233; doi:10.1038/s41598-018-22026-0)
Supplement: Supplementary file 1 — Supplementary Information [file 41598_2018_22026_MOESM1_ESM.pdf]

Supplementary information

PV1, a novel *Plasmodium falciparum* merozoite dense granule protein, interacts with exported protein in infected erythrocytes

Masayuki Morita[1], Hikaru Nagaoka[1], Edward H. Ntege[1], Bernard N. Kanoi[1], Daisuke Ito[1#], Takahiro Nakata[1], Ji-Won Lee[2], Kazuaki Tokunaga[3], Tadahiro Iimura[2,4], Motomi Torii[5], Takafumi Tsuboi[1] and Eizo Takashima[1*]

[1]Division of Malaria Research, Proteo-Science Center, Ehime University, Matsuyama, Ehime, Japan; [2]Division of Bio-Imaging, Proteo-Science Center, Ehime University, Shitsukawa, Toon, Ehime, Japan; [3]Nikon Instech CO., LTD., Shinagawa, Tokyo, Japan; [4]Division of Analytical Bio-Medicine, Advanced Research Support Center, Ehime University, Shitsukawa, Toon, Ehime, Japan; [5]Division of Molecular Parasitology, Proteo-Science Center, Ehime University, Shitsukawa, Toon, Ehime, Japan.

#Present address: Tottori University, Faculty of Medicine

Department of Medical Zoology 86 Nishi-cho Yonago, Tottori 683-8503, Japan

*Correspondence author: Eizo Takashima

Address: Division of Malaria Research, Proteo-Science Center, Ehime University, Japan, 3 Bunkyo-cho, Matsuyama, Ehime 790-8577, Japan.

Tel., +81-89-927-9939; E-mail, eizo.takashima.mz@ehime-u.ac.jp

Figure legends

Figure S1

Subcellular localization of PfPV1 in ring and trophozoite stage, by confocal microscopy: PfPV1 (green) was co-stained with RAP1 (red) as a PV marker, RESA (red) as PV and RBC membrane marker; and SBP1 (red) as Maurer's cleft marker. The parasite nucleus was stained with DAPI (blue). Scale bar, 5μm.

Figure S2

Analysis of interaction between PV1, RAP1 and PTP5: a) Results of immunoprecipiation experiment using rabbit anti-PfPV1 antibody, the membrane was probed with anti-PTP5 antibody, as shown in Fig.4b b) Immunoprecipitation experiment with rabbit anti-PTP5 antibody; To detect PV1, the immunoblot was probed with mouse anti-PV1 antibody. c) The figures show results of immunoprecipitation experiment with rabbit anti-PfPV1 antibody. In the Western blot analysis, mouse anti-RAP1 antibody was used to detect RAP1. In a, b and c, parasite lysate was derived from 10^8 trophozoite-/schizont-rich parasite pellet, and a corresponding sample immunoprecipitated with rabbit anti-GST antibody (GST IP) included as a negative control. To confirm reproducibility, all immunoprecipitation experiments were validated in three independent experiments. .

Figure S3

Characterization of PF3D7_0801000 as a PfPV1 interacting protein: a) Schematic representation of the primary structure of PF3D7_0801000 and PF3D7_0801000N. b) Purified recombinant GST-tagged PF3D7_0801000N resolved in SDS-PAGE and visualized by CBB staining, c) Immunoprecipitation of PF3D7_0801000 with rabbit anti-PfPV1 antibody, the immunoblot was probed with rabbit anti-PF3D7_0801000 antibody. To confirm reproducibility, three independent experiments were carried out. d) SPR analysis of interaction between recombinant proteins His-tagged PfPV1 (as ligand) and PF3D7_0801000N (as analyte). The analyte was applied in multiple concentrations; 2.4, 12, 60, 300, and 1500 nM. The black line represents line of fit while purple line is the SPR sensorgram.

Figure S4

Immunoprecipitation of PTEX components with PfPV1: For Western blot analysis we used mouse anti-EXP2 or HSP101 antibody to detect PTEX components. Lysate was derived from 10^8 trophozoite-/schizont-rich parasite pellet. GST IP was prepared with sample immunoprecipitated with rabbit anti-GST antibody and was included as a negative control. All immunoprecipitation experiments were validated by three independent experiments.

Figure S5

Immunoprecipitation experiments for HA tagged PTP5 proteins: a) We used rabbit anti-PfPV1 antibody for immunoprecipiation experiment and anti-HA antibody for subsequent Western blot analysis. Lane 1; Protein molecular weight marker. Lane 2; PTP5FL-HA parasite lysate immunoprecipitated with rabbit anti-GST antibody (Negative control). Lane 3; PTP5FL-HA parasite lysate immunoprecipitated with anti-PfPV1 antibody. Lane 4; PTP5ΔPHR-HA parasite lysate immunoprecipitated with rabbit anti-GST antibody (Negative control). 5; PTP5ΔPHR-HA parasite lysate immunoprecipitated with anti-PfPV1 antibody. The asterisks indicate signals corresponding to PTP5FL-HA and PTP5ΔPHR-HA. To confirm reproducibility, three independent experiments were carried out.

b) Immunoprecipitation experiment was performed with 10 μg/ml of rat anti-HA monoclonal antibody (clone 3F10, IgG), and rabbit anti-PV1 antibody applied for subsequent Western blot analysis. Immunoprecipitation sample prepared with Rat IgG (10 μg/ml; Sigma, Cat.no. I4131) was used as a negative control. Left panel represents PTP5FL-HA parasite lysate while right panel represents PTP5ΔPHR-HA parasite lysate derived from 10^8 trophozoite-/schizont-rich parasite pellet. To confirm reproducibility, three independent experiments were carried out.

Movie S1

3D-SIM result of PTP5FL-HA. Subcellular localization of PTP5FL-HA (green), EXP2 (red) and PfPV1 (cyan) as observed with 3D-SIM.

Movie S2

3D-SIM result of PTP5ΔPHR-HA. Subcellular localization of PTP5ΔPHR-HA (green), EXP2 (red) and PfPV1 (cyan) as observed with 3D-SIM.

Table S1 Information of the PCR primers

Name	Sequence
for pEU vector	
PV1F	GAGAGAGACTCGAGGTGGTGGCCCCTAAGAGTGCTGAG
PV1R	GAGAGAGAGCGGCCGCCTAGCTCGATATTGGTGTGTTTTGATCATTTC
PTP5F	GAGAGAGACTCGAGACATATAATAATTATACTTATAATGGTTATAACAATGACG
PTP5R	GAGAGAGAGCGGCCGCTCATTATTTTAATTTCTTTTGAGATCTACCATATAAACG
PTP5Tr1R	GAGAGAGAGCGGCCGCTCAAGATGATGTTTTACCCCCACTT
PTP5Tr2F	GAGAGAGACTCGAGTCCCCTCTTTTAAAAAGCGAAAC
0801NF	GAGAGAGACTCGAGGGAGCGAATCCAGAATCCATTAGTTTAGG
0801NR	GAGAGAGAGCGGCCGCCTAATGGTGATGGTGATGGTGAAGGTTCTTTTTATTAAACTTGGAAGGTTTTATAATATCA
for pD vector	
PTP5FL-F	GAGAGAGCGGCCGCATGGAAAACATAATAAACAAGAAGAATACG
PTP5FL-R	TCTCTCCTGCAGCTTTTAATTTCTTTTGAGATCTACCATATAAACG
PTP5ΔPHR-R	TCTCTCCTGCAGCAGATGATGTTTTACCCCCACTTGA

Fig. S1

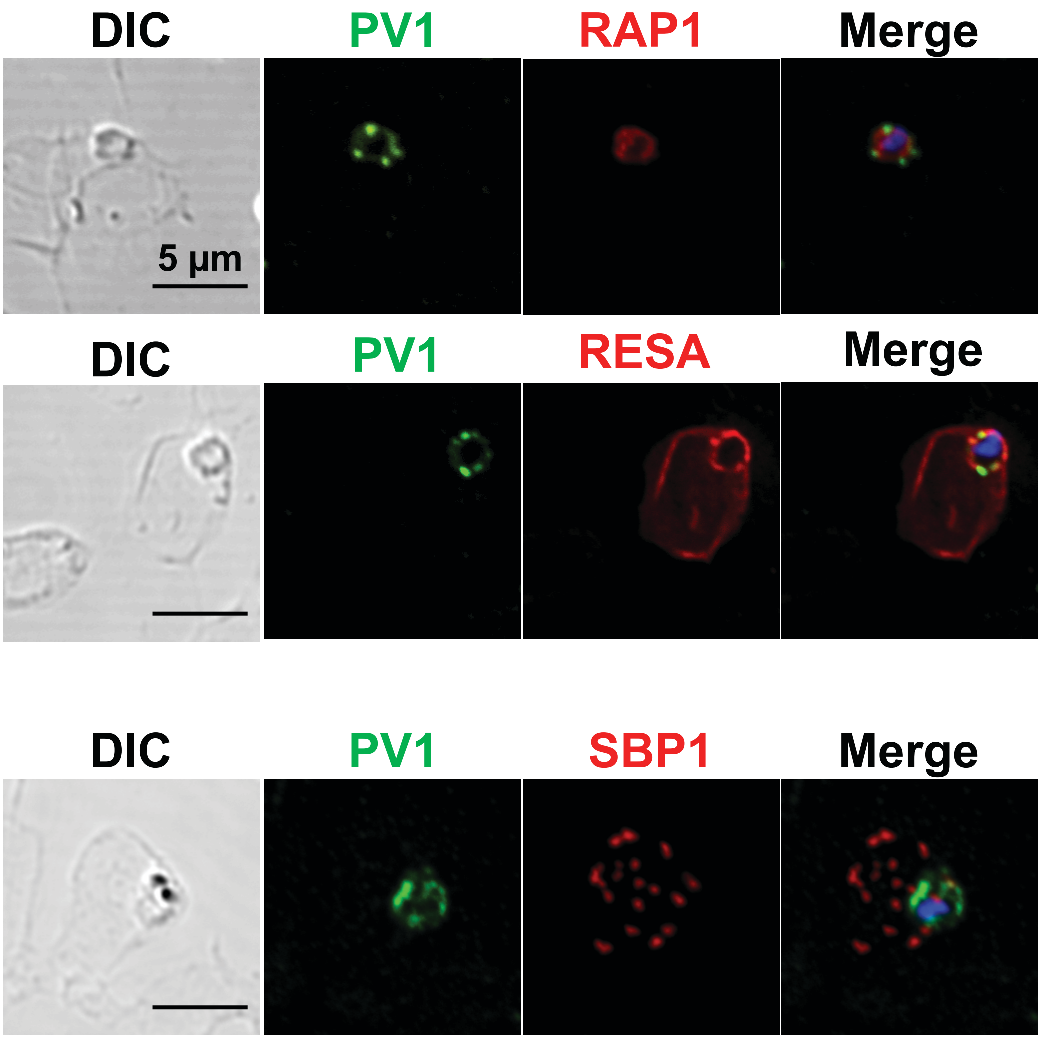

Fig. S2

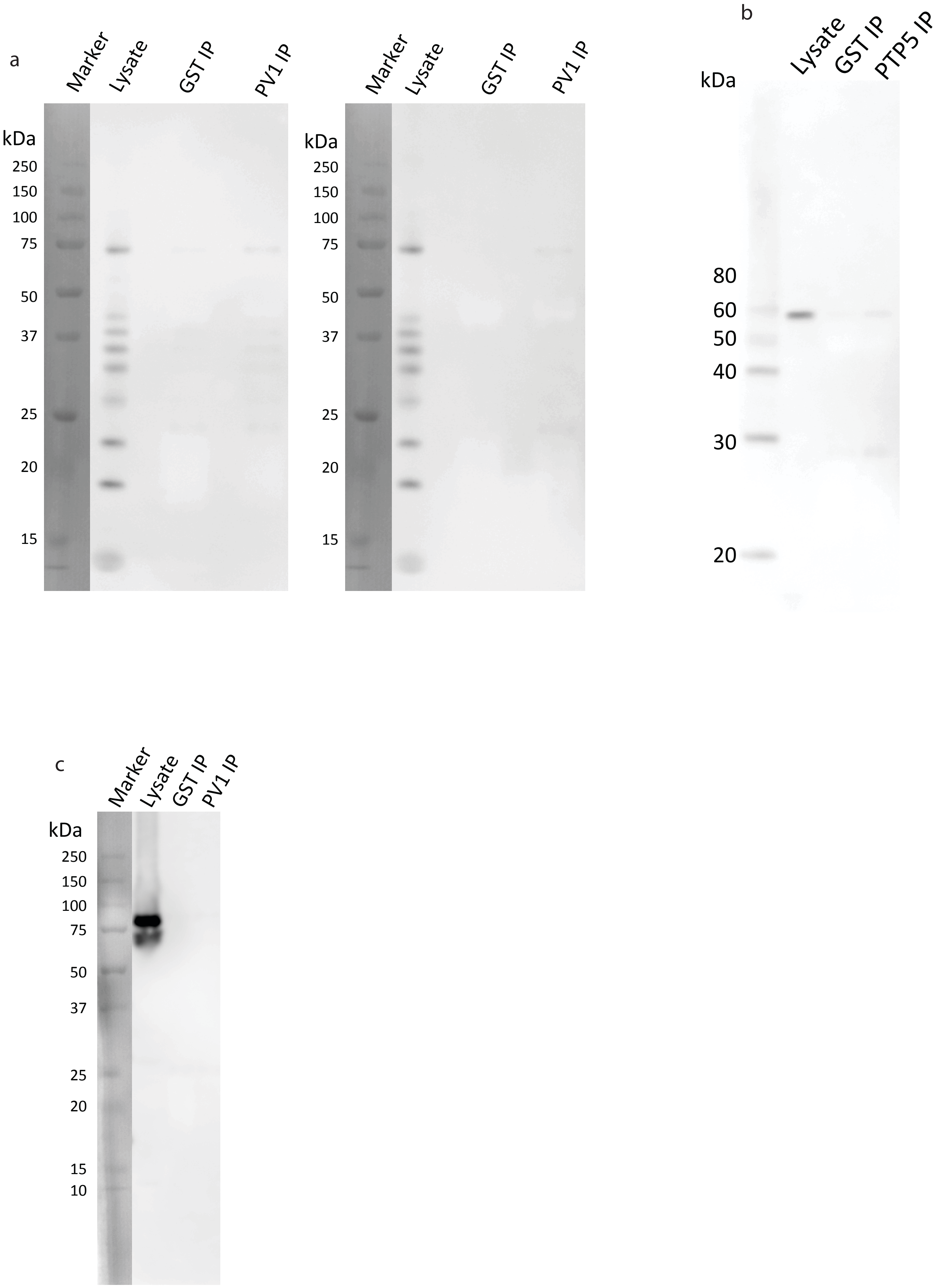

a
Marker
Lysate
GST IP
PV1 IP
kDa
250
150
100
75
50
37
25
20
15
Marker
Lysate
GST IP
PV1 IP
kDa
250
150
100
75
50
37
25
20
15
b
Lysate
GST IP
PTP5 IP
kDa
80
60
50
40
30
20
c
Marker
Lysate
GST IP
PV1 IP
kDa
250
150
100
75
50
37
25
20
15
10

Fig.S3

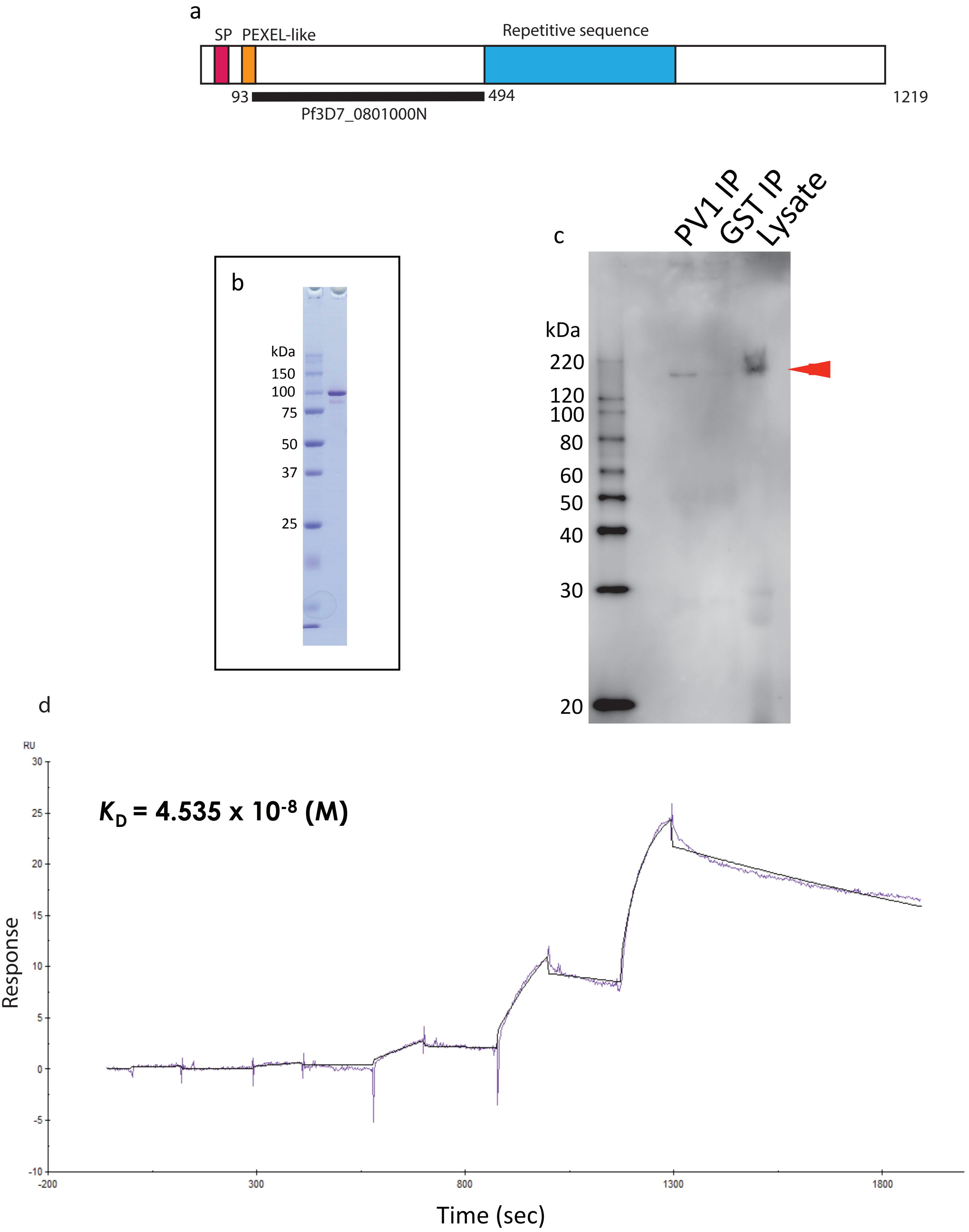
a
SP
PEXEL-like
Repetitive sequence
93
494
1219
Pf3D7_0801000N
b
kDa
150
100
75
50
37
25
c
PV1 IP
GST IP
Lysate
kDa
220
120
100
80
60
50
40
30
20
d
RU
K_D = 4.535 x 10^{-8} (M)
Response
Time (sec)
30
25
20
15
10
5
0
-5
-10
-200
300
800
1300
1800

Fig. S4

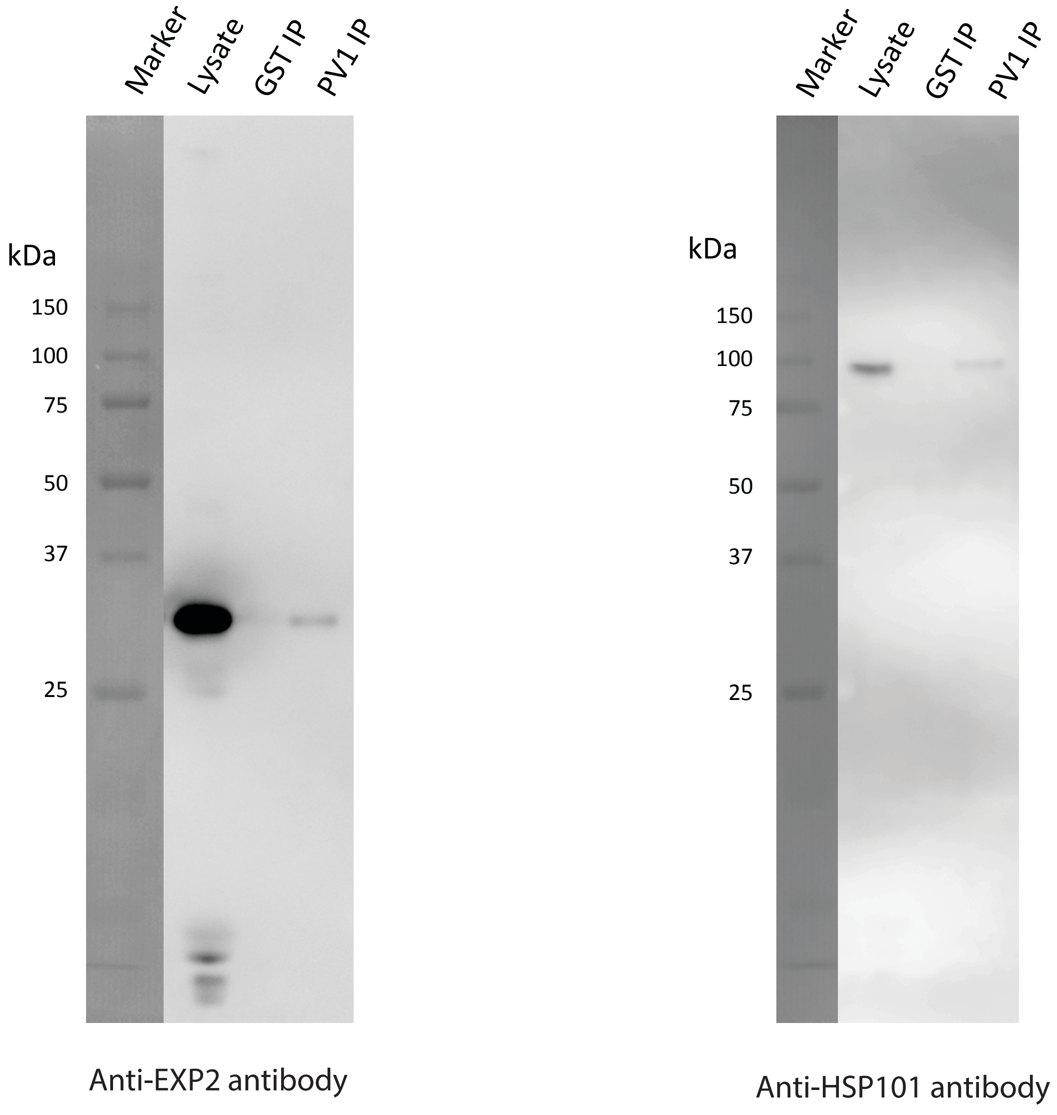

Marker
Lysate
GST IP
PV1 IP
kDa
150
100
75
50
37
25
Anti-EXP2 antibody
Marker
Lysate
GST IP
PV1 IP
kDa
150
100
75
50
37
25
Anti-HSP101 antibody

Fig.S5

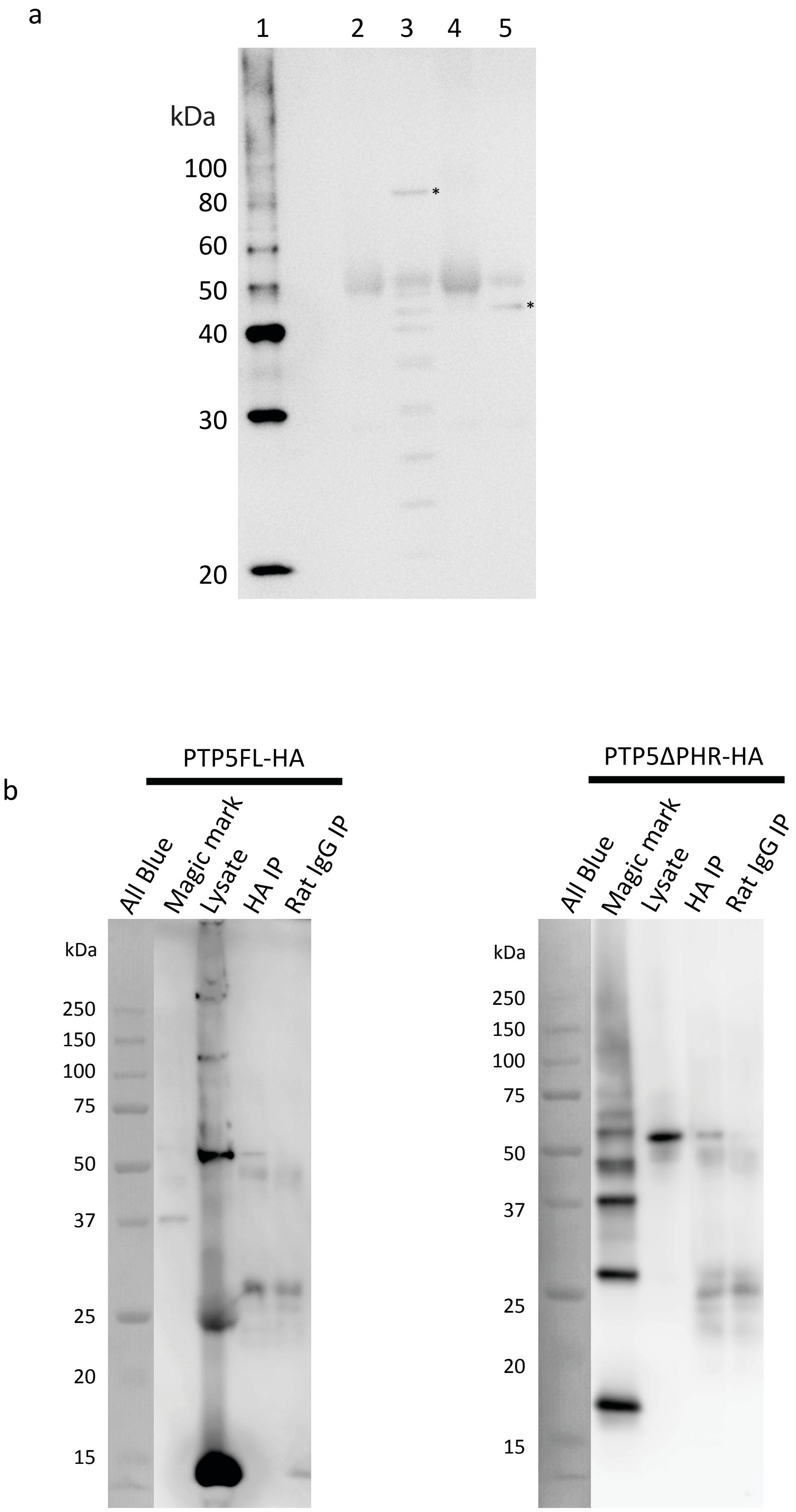
a
1
2
3
4
5
kDa
100
80
60
50
40
30
20
*
*
b
PTP5FL-HA
All Blue
Magic mark
Lysate
HA IP
Rat IgG IP
kDa
250
150
100
75
50
37
25
20
15
PTP5ΔPHR-HA
All Blue
Magic mark
Lysate
HA IP
Rat IgG IP
kDa
250
150
100
75
50
37
25
20
15
